# Scavenging acrolein with 2-HDP preserves neurovascular integrity in a rat model of diabetic retinal disease

**DOI:** 10.1007/s00125-025-06515-2

**Published:** 2025-08-15

**Authors:** Josy Augustine, Evan P. Troendle, Thomas Friedel, Caolan Baldwin, Eimear M. Byrne, Sadaf Ashraf, Paul Canning, Corey A. McAleese, Adam G. Rollo, Peter Barabas, Timothy J. Lyons, Martin B. Ulmschneider, Alan W. Stitt, Tim M. Curtis

**Affiliations:** 1 Wellcome-Wolfson Institute for Experimental Medicine, Queen’s University, Belfast, UK; 2 Department of Chemistry, King’s College London, London, UK; 3 Medway School of Pharmacy, Universities of Kent and Greenwich, Chatham, UK; 4 Division of Endocrinology and Diabetes, Medical University of South Carolina, Charleston, SC USA; 5 Diabetes Free South Carolina, BlueCross BlueShield of South Carolina, Columbia, SC USA

**Keywords:** 2-Hydrazino-4,6-dimethylpyrimidine (2-HDP), Acrolein, Diabetic retinal disease (DRD), Electroretinography (ERG), Molecular dynamics (MD) simulations, Neurodegeneration, Neurovascular unit (NVU), *N*ε-(3-formyl-3,4-dehydropiperidino)lysine (FDP-Lys), Retinal inflammation, Vascular pathology

## Abstract

**Aims/hypothesis:**

Diabetic retinal disease (DRD) is characterised by progressive neurovascular unit (NVU) dysfunction, often occurring before visible microvascular damage. Our previous studies suggested that the accumulation of acrolein (ACR)-derived protein adducts on retinal Müller cells and neuronal proteins may contribute to NVU dysfunction in diabetes, although this has yet to be directly tested. In this study, we evaluated the effects of the novel ACR-scavenging drug 2-hydrazino-4,6-dimethylpyrimidine (2-HDP) on retinal NVU dysfunction in experimental diabetes and explored its potential for systemic delivery in humans.

**Methods:**

Sprague Dawley rats were divided into three groups: non-diabetic rats; streptozocin (STZ)-induced diabetic rats; and STZ-induced diabetic rats treated with 2-HDP in their drinking water throughout the duration of diabetes. Endpoint measures were taken at varying time points, ranging from 1 to 6 months post-diabetes induction. Retinal function and structure were evaluated using electroretinography (ERG) and spectral-domain optical coherence tomography (SD-OCT). Retinal vessel calibre, BP and vasopermeability (assessed by Evans Blue leakage) were also monitored. Immunohistochemistry was employed to assess retinal neurodegenerative and vasodegenerative changes, while cytokine arrays were used to investigate the effect of 2-HDP on diabetes-induced retinal inflammation. The accumulation of the ACR–protein adduct *N*ε-(3-formyl-3,4-dehydropiperidino)lysine (FDP-Lys) in human diabetic retinas was analysed. Computational chemistry simulations were performed to predict 2-HDP’s passive permeability properties and its potential for systemic delivery.

**Results:**

2-HDP treatment had no effect on blood glucose, body weight, water intake, HbA_1c_ levels or BP in diabetic rats (*p*>0.05). However, it protected against retinal FDP-Lys accumulation (*p*<0.05) and neurophysiological dysfunction, preserving ERG waveforms at 3 and 6 months post-diabetes induction (*p*<0.05 to *p*<0.001 for scotopic for a-wave, b-wave and summed oscillatory potentials). SD-OCT imaging revealed that 2-HDP prevented retinal thinning at 3 months (*p*<0.01) and protected against synaptic dysfunction, as evidenced by preserved synaptophysin expression (*p*<0.01 and *p*<0.001 for inner and outer plexiform layers, respectively). It also prevented neurodegeneration by maintaining retinal ganglion cells, amacrine cells, bipolar cells, and photoreceptors (*p*<0.05 to *p*<0.01). In addition, 2-HDP prevented retinal arteriolar dilation (*p*<0.01), reduced microvascular permeability (*p*<0.05) and attenuated microvascular damage, as indicated by preserved pericyte numbers and reduced acellular capillary formation (*p*<0.05). Mechanistically, 2-HDP inhibited microglial activation (*p*<0.05), suppressed the upregulation of proinflammatory molecules associated with NVU dysfunction in the diabetic retina (*p*<0.05 to *p*<0.001) and preserved the expression of the Müller cell glutamate-handling proteins, glutamate aspartate transporter 1 and glutamine synthetase (*p*<0.05 to *p*<0.01). FDP-Lys accumulation was observed in post-mortem human retinas from individuals with type 2 diabetes (*p*<0.05), in a pattern that was similar to that in the rat model of diabetes. Molecular dynamics simulations showed that the neutral form of 2-HDP readily crosses cell membranes, with enhanced permeation in the presence of ACR, highlighting its potential for systemic delivery.

**Conclusions/interpretation:**

2-HDP protects against retinal NVU dysfunction in diabetic rats by reducing FDP-Lys accumulation, preserving neuroretinal function and preventing microvascular damage, independent of glycaemic control. These results, combined with evidence from human diabetic retinas and molecular dynamics simulations, support 2-HDP’s potential as a promising therapeutic agent for DRD, warranting further preclinical and clinical investigation.

**Graphical Abstract:**

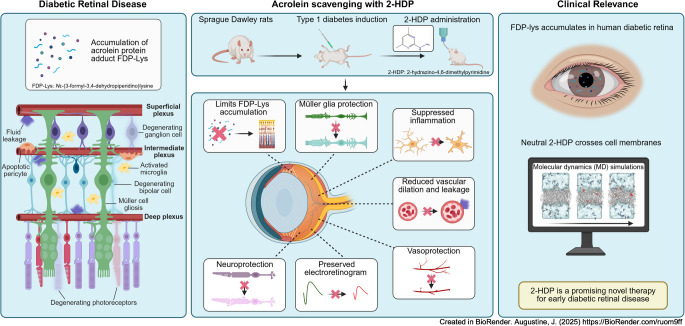

**Supplementary Information:**

The online version contains peer-reviewed but unedited supplementary material available at 10.1007/s00125-025-06515-2.


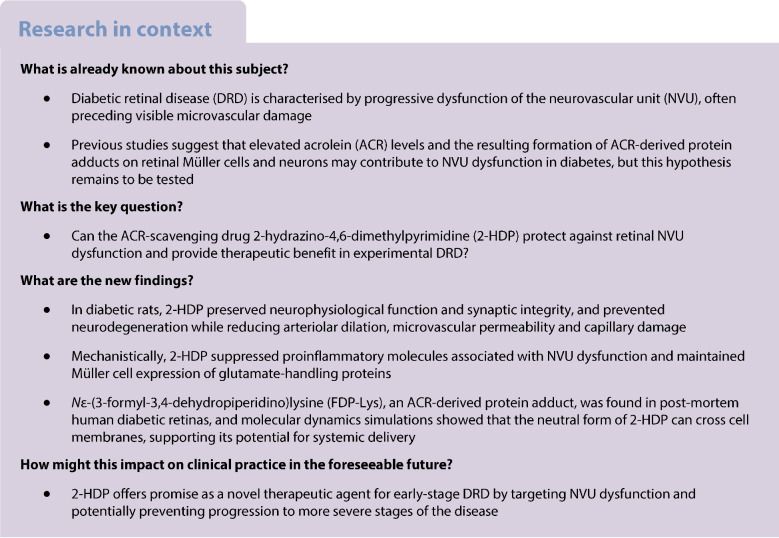


## Introduction

Diabetic retinal disease (DRD) is a leading cause of blindness among working-age adults [1]. Despite advances in systemic management of diabetes and ocular treatments such as anti-vascular endothelial growth factor (VEGF) therapy, laser photocoagulation and vitreous surgery, many affected individuals still progress to sight-threatening complications, including diabetic macular oedema (DMO) and proliferative diabetic retinopathy (PDR) [2–5]. Current therapies primarily target microvascular damage at advanced stages of disease and are limited by their invasiveness, need for repeated administration and incomplete efficacy, highlighting the need for new treatment strategies that address earlier pathogenic events.

Recent evidence indicates that diabetes affects not only the retinal microvasculature but also the entire neurovascular unit (NVU), comprising vascular, glial, neuronal and immune components [6, 7]. Neuronal dysfunction and retinal neurodegeneration occur early in DRD, often preceding detectable vascular pathology [7–9]. In addition, Müller glia, which maintain retinal homeostasis and support both neurons and vessels, undergo reactive gliosis in diabetes, contributing to neuroinflammation, breakdown of the inner blood–retina barrier (iBRB), retinal neovascularisation and neuronal damage [10–13]. They also regulate extracellular glutamate, and their dysfunction can result in glutamate excitotoxicity, further exacerbating neuronal damage in DRD [14–16]. Therapeutic strategies capable of protecting the entire retinal NVU during diabetes could therefore represent a significant advance in the prevention and treatment of DRD.

To develop effective strategies for preserving retinal NVU integrity during diabetes, it is essential to understand the mechanisms driving its disruption. Our previous work in streptozocin (STZ)-induced diabetic rats suggests that elevated levels of the lipid peroxidation product acrolein (ACR) and accumulation of the ACR-derived protein adduct *N*ε-(3-formyl-3,4-dehydropiperidino)lysine (FDP-Lys) on retinal Müller cell and neuronal proteins contribute significantly to NVU dysfunction in diabetes. FDP-Lys accumulation was detected in Müller glia within 1–2 months of experimental diabetes, with progressive accumulation in retinal ganglion cells (RGCs) and inner nuclear layer (INL) neurons as the disease advanced [17]. Notably, this accumulation was selective, with no increases in other lipoxidation-derived protein adducts, such as dihydropyridine-lysine, or adducts from 4-hydroxynonenal and 4-hydroxyhexanal [17]. ACR is well-established as a neurotoxic and gliotoxic agent in retinal and brain cells [18–20], while intracellular FDP-Lys accumulation induces cytotoxicity by covalently binding thiols, promoting protein–protein crosslinking [21] and depleting glutathione (GSH), thereby triggering oxidative stress [22]. Consistent with this, we previously showed that FDP-Lys-modified proteins in cultured human retinal Müller cells, even without exogenous ACR, induce oxidative stress, upregulate proinflammatory factors and promote programmed cell death [17]. Collectively, these findings suggest that targeting ACR and FDP-Lys formation could offer an effective means of protecting the NVU during the early stages of DRD.

We previously identified 2-hydrazino-4,6-dimethylpyrimidine (2-HDP) as a novel and potent ACR scavenger that outperformed other known ACR-scavenging compounds in cell-free ELISA assays [23]. Oral administration of 2-HDP to diabetic rats for 3 months prevented retinal FDP-Lys accumulation, Müller cell gliosis, oxidative stress, proinflammatory signalling and microglial activation [23]. Given the pivotal role of Müller cells in NVU dysfunction in DRD, these findings indicate that 2-HDP may offer a promising therapeutic approach to prevent NVU dysfunction and its neuronal and vascular complications. However, this potential has not yet been directly investigated.

This study aimed to determine whether 2-HDP prevents retinal NVU dysfunction in STZ-induced diabetic rats. We evaluated its effects on retinal function, synaptic integrity, neurodegeneration and vascular pathology, alongside mechanistic analyses of its protective effects. To explore its clinical potential, we examined FDP-Lys accumulation in human diabetic retinas and used computational chemistry to evaluate 2-HDP’s passive permeability properties to assess its suitability for systemic delivery in humans.

## Methods

### Diabetic rat model

Male Sprague Dawley rats weighing 250–400 g (Inotiv, Bicester, UK) were randomly allocated into one of three groups: (1) non-diabetic rats; (2) streptozocin (STZ)-induced diabetic rats; and (3) STZ-induced diabetic rats treated with 2-HDP. Rats assigned to diabetic groups received i.p. injections of STZ (Sigma-Aldrich, Gillingham, UK) at a dose of 50 mg/kg, dissolved in 0.1 mol/l sodium citrate buffer (pH 4.5). Blood glucose levels were measured 1 week after STZ injection using an Alphatrak Glucometer (Zoetis, Leatherhead, UK). Rats with blood glucose levels exceeding 15 mmol/l were considered to be diabetic and used in the study. Non-diabetic control rats received sham injections of citrate buffer alone. All rats were housed in conventional, pathogen-free animal facilities with a 12 h light–dark cycle and provided with ad libitum access to food and water. Diabetic rats were administered Caninsulin (MSD Health, London, UK) at a dose of 10 U/kg, given twice weekly to manage the severity of hyperglycaemia. The 2-HDP-treated group had 100 mg/l of 2-HDP (Fluorochem, Hadfield, UK) directly dissolved in their drinking water 1 week after diabetes induction, with daily monitoring of water consumption to determine the administered dose [23]. Measurements of body weight, blood glucose and HbA_1c_ (PTS Diagnostics, Whitestown, IN, USA) were obtained at 1, 3 and 6 months post-diabetes induction. No adverse events were associated with the 2-HDP intervention.

### BP

BP measurements were performed on conscious rats from all experimental groups at 3 months after diabetes induction using tail cuff plethysmography (ML125 NIBP System; AD Instruments, Oxford, UK). Prior to the experiments, a habituation period of 7 days was provided to allow the rats to acclimatise to the plethysmography procedure. Triplicate measurements of each animal’s BP were taken and, subsequently, the mean systolic, diastolic and mean arterial BPs were calculated.

### Electroretinography

Scotopic electroretinograms (ERGs) were performed at intervals of 1, 3 and 6 months following diabetes induction using an Espion Visual Electrophysiology System (Diagnosys, Cambridge, UK). Rats were subjected to overnight dark adaptation, followed by anaesthesia using sodium pentobarbital (50 mg/kg) and pupil dilation with 1% atropine and 2.5% phenylephrine eye drops (Bausch & Lomb, London, UK). Prior to ERG recording, Viscotears Liquid Gel (Bausch & Lomb) was applied to maintain ocular moisture. ERGs were recorded for both eyes. Rodent corneal electrodes were carefully positioned, with the reference and ground electrodes located on the forehead and tail, respectively. The a- and b-wave amplitudes were assessed over a range of increasing light intensities (0.008–25 cd s m^−2^). ERG signals were averaged from five responses at each intensity level, with interstimulus intervals of 15 s (0.008 cd s m^−2^), 30 s (0.025, 0.08, 0.25 cd s m^−2^) 45 s (0.8 cd s m^−2^), 60 s (2.5 cd s m^−2^) or 120 s (8, 25 cd s m^−2^). Oscillatory potentials (OPs) were extracted by applying a high-pass filter and recording at a flash intensity of 25 cd s m^−2^. Summed OP amplitudes were subsequently calculated from wavelets 2–5.

### Spectral-domain optical coherence tomography

Rats were anaesthetised and their pupils were dilated as described above. Subsequently, spectral-domain optical coherence tomography (SD-OCT) images, encompassing a 30° field of view, were acquired at 1 and 3 months after the induction of diabetes using a Spectralis-Heidelberg OCT system (Heidelberg Engineering, Heidelberg, Germany). Retinal thickness was measured in both eyes 1500 μm from the optic disc across all four retinal quadrants (nasal, temporal, superior and inferior) and the results were averaged for statistical analysis.

### Retinal vessel calibre

Rats were anaesthetised and their pupils were dilated as previously outlined. Retinal fundus images were obtained from all experimental groups at 3 months post-diabetes induction using a Micron IV rat fundus camera (Phoenix Research Laboratories, Bend, OR, USA). The fundus images, centred on the optic nerve, were processed and analysed using Automated Retinal Image Analyser (ARIA) v1.0 Software [24]. The optic disc was manually demarcated and arterioles traversing a predefined region of interest (ROI) located at a distance ranging from one-half to two-and-a-half disc diameters from the optic disc were quantified. To ensure consistent vessel detection and precise calculation of mean diameters within the ROI, the four largest arterioles in each image were selected for analysis. Data from both eyes were averaged for each rat, and these values were subsequently used for statistical analysis.

### Immunohistochemistry

#### Human retinas

For immunohistochemistry (IHC) examination, post-mortem human retinas were obtained from individuals with type 2 diabetes and non-diabetic individuals of similar age via the National Disease Research Interchange (Philadelphia, PA, USA), with each group comprising three individuals. Demographic information about the sample donors can be found in electronic supplementary material (ESM) Table 1. Antigen retrieval and immunolabelling were performed on retinal sections (14 μm thick) as previously described [25].

Details of the primary and secondary antibodies, including their dilutions in blocking buffer (10% normal donkey serum, 0.3% Triton X-100 and 0.1% NaN_3_ in PBS), are provided in ESM Table 2.

Retinal sections were cover-slipped with DAPI-containing Vector Shield (Vector Labs, Peterborough, UK) and imaged using a Leica SP5 confocal laser scanning microscope (Leica Microsystems, Milton Keynes, UK).

#### Rat retinas

Eyes were harvested 6 months after diabetes induction and were fixed in 4% wt/vol. paraformaldehyde (Sigma-Aldrich) for 1 h at room temperature. The processing and immunolabelling of rat retinal whole mounts and cryosections followed established protocols as previously outlined [25]. ESM Table 3 lists the primary and secondary antibodies used, along with their dilutions in blocking buffer containing normal donkey serum (10% for cryosections, 2% for flatmounts; Sigma-Aldrich) and 0.5% Triton X-100 in PBS (Sigma-Aldrich). To visualise cell nuclei, DAPI (Vector Laboratories) was employed as a counterstain. For the detection of isolectin binding, isolectin-B4 from *Bandeiraea simplicifolia* (1:200; biotin conjugate; Sigma-Aldrich; catalogue L2140) was used, followed by incubation with streptavidin conjugated to Alexa Fluor 647 (1:200; Invitrogen; catalogue S21374). Confocal images were acquired using a Leica SP5, Leica SP8 (Leica Microsystems), or Nikon C1 (Nikon, Kingston upon Thames, UK) laser scanning confocal microscope.

#### Analysis of IHC images

Confocal parameters were kept consistent throughout each experiment, and images were processed and analysed using Fiji ImageJ version 1.54m software [26]. For IHC, one eye per rat or human was analysed. Retinal data were collected from three sections per eye for both rats and humans. For rats, 5–8 images per section were obtained and averaged per eye for statistical analysis, while for humans, 3–6 images per section were acquired and similarly averaged per eye. The number of dendritic and amoeboid microglial cells in the ganglion cell layer, inner plexiform layer and outer plexiform layer of rat retinal sections was quantified using the multi-point tool in Fiji software. Dendritic microglia were identified by their small somas and at least two thin, highly branched processes exceeding the soma diameter, while amoeboid microglia exhibited enlarged, coarse somas with fewer than three thick processes shorter than the soma diameter [27, 28]. Imaging of rat whole-mount retinal preparations focused on the superficial vascular plexus, with two images from each of the four retinal quadrants averaged per eye. To minimise subjective bias, the Fiji blind analysis tool was used to ensure that the experimenter was unaware of the group identity while analysing all IHC data in this study.

### Western blotting

Retinas were extracted from rats after 3 months of diabetes duration and lysed in RIPA buffer with added protease inhibitors (Thermo Fisher Scientific). The resulting supernatant fractions were clarified by centrifugation at 12,000 *g* for 15 min (4°C) and protein concentration were determined using a bicinchoninic acid protein assay kit (Thermo Fisher Scientific).

Protein samples (30 μg) were separated on 10% SDS–polyacrylamide gels, transferred to PVDF membranes and probed with antibodies listed in ESM Table 4, using a blocking buffer of 5% BSA in Tris-buffered saline with 0.1% Tween-20 (TBST; Sigma-Aldrich).

After washing, the membranes were incubated with suitable horseradish-peroxidase-conjugated or IRDye 800 secondary antibodies (ESM Table 4). Immunoreactive bands were visualised using enhanced chemiluminescence or infrared imaging with a G:BOX XX6 chemiluminescence system (Syngene, Cambridge, UK) or an LI-COR Odyssey Infrared Imaging System (LI-COR Biosciences, Lincoln, NE, USA). Densitometry was used for immunoblot quantification, and protein expression was normalised to β-actin levels.

### Cytokine array

After 3 months of diabetes, levels of retinal inflammatory cytokines were assessed in each of the experimental groups using a Rat Cytokine Antibody Array Kit (Abcam, Cambridge, UK; ab133992) according to established procedures [25]. A total of 300 μg of protein from six retinas was applied per array. The array images were analysed using Fiji ImageJ version 1.54m software [26]. Background staining was subtracted and the relative intensity of each spot was normalised to positive control spots on each array. The integrated density of duplicate spots for each cytokine was quantified and the mean calculated for each membrane. The cytokine array experiment was performed using pooled retinas from six animals per experimental group, with two or three technical replicates per group.

### Retinal vasopermeability

After 3 months of diabetes, the assessment of blood–retina barrier disruption in each experimental group was conducted by quantifying albumin leakage into the retina using the Evans Blue assay [25]. The concentration of dye in the extracts was determined from a standard curve of Evans Blue in formamide, and Evans Blue leakage was quantified using the formula detailed in [29].

### Computational chemistry

Computational chemistry techniques were employed to assess the permeability properties of 2-HDP across cell membranes. Cheminformatics analysis of microspecies abundance at pH 7.0 was performed using Chemicalize (https://chemicalize.com/, developed by ChemAxon https://chemaxon.com/, accessed 6 May 2021). All-atom equilibrium molecular dynamics (MD) simulations were conducted using an established human blood–brain barrier (BBB) screening model to predict the transport behaviour of 2-HDP under physiological conditions [30, 31]. The simulations examined both protonated and neutral forms of 2-HDP to account for potential permeability changes associated with its ionisation state. In addition, given that ACR levels are elevated in the diabetic retina, this molecule was included in supplementary simulations to evaluate its impact on 2-HDP transport. For comparison, a benchmark simulation of caffeine was also conducted. Full methodological details and key parameters for reproducing the MD simulations and analyses are provided in the ESM Methods.

### Statistics

Data are presented as bar plots displaying individual data points along with the mean ± SEM. Statistical analysis was performed on biological replicates, with technical replicates averaged to represent each biological replicate. Normality was assessed using the Shapiro–Wilk test. Comparisons were made using two-tailed Student’s *t* tests or one-way/two-way ANOVA with Bonferroni’s post hoc correction for multiple comparisons. To account for the large number of comparisons in the cytokine arrays, corrections for multiple comparisons were applied using the Benjamini–Hochberg False Discovery Rate method [32]. *p*<0.05 was considered statistically significant. In the figure captions, all *p* values from ANOVA tests refer to post hoc comparisons. Statistical analyses were conducted using GraphPad Prism software (v10.3.1).

### Study approvals

Human studies were approved by the Research Ethics Committee at Queen’s University Belfast and conducted in accordance with the principles of the Declaration of Helsinki. All animal procedures were approved by the Queen’s University Belfast Animal Welfare and Ethical Review Body and carried out in accordance with the Association for Research in Vision and Ophthalmology (ARVO) Statement for the Use of Animals in Ophthalmic and Vision Research. Animals received humane care in compliance with the National Institutes of Health (NIH) Guide for the Care and Use of Laboratory Animals (8th edition, 2011). The work adhered to the Department of Health, Social Services, and Public Safety (DHSSPS) project licenses PPL2786 and PPL2888.

## Results

### Metabolic and physiological characteristics of experimental animal groups

**Fig. 1:**
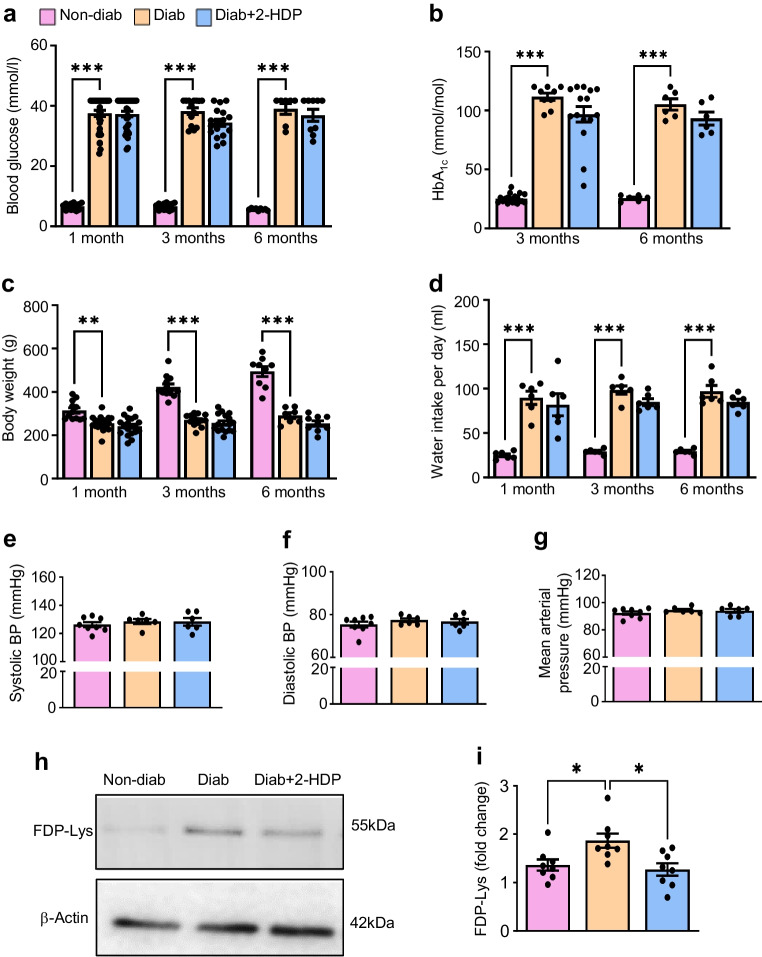
Metabolic and physiological characteristics of the experimental groups of rats. (**a**–**g**) Bar charts show the effects of 2-HDP on diabetes-induced changes in blood glucose levels (**a**), HbA_1c_ levels (**b**), body weight gain (**c**), water intake (**d**) and BP variables (**e**–**g**). *n*=6–34 animals per experimental group. (**h**) Representative western blot showing FDP-Lys levels in retinas from non-diabetic, diabetic and 2-HDP-treated diabetic rats after 3 months of diabetes. Each lane represents an individual rat. (**i**) Bar graph showing that 2-HDP treatment reduces FDP-Lys accumulation in the diabetic retina. *n*=8 rats per experimental group. *p<0.05, **p<0.01 and ***p<0.001 for the indicated comparisons. Diab, diabetic

Blood glucose, body weight and water intake were monitored at 1, 3 and 6 months post-diabetes induction, with HbA_1c_ assessed at 3 and 6 months. Diabetic and 2-HDP-treated diabetic rats displayed elevated blood glucose and HbA_1c_ levels compared with non-diabetic controls, with no significant difference between the treated and untreated diabetic groups (Fig. 1a, b). While non-diabetic rats consistently gained weight, the weights of diabetic and 2-HDP-treated diabetic rats remained constant throughout the study (Fig. 1c). Diabetic and 2-HDP-treated diabetic rats showed increased water intake compared with non-diabetic controls (Fig. 1d). 2-HDP did not affect fluid consumption in diabetic rats. The mean daily dose of 2-HDP was determined as 40 mg/kg using the recorded fluid intake data. Measurement of systolic BP, diastolic BP and mean arterial pressure at 3 months revealed no differences between the experimental groups, indicating that 2-HDP has no impact on BP in diabetic rats (Fig. 1e–g). To validate our previous findings demonstrating that 2-HDP inhibits FDP-Lys accumulation in the diabetic retina [23], we conducted western blot analysis of protein samples from each experimental group. As expected, the administration of 2-HDP led to decreased retinal FDP-Lys accumulation after 3 months of diabetes duration (Fig. 1h, i).

### 2-HDP protects against neurophysiological dysfunction

**Fig. 2:**
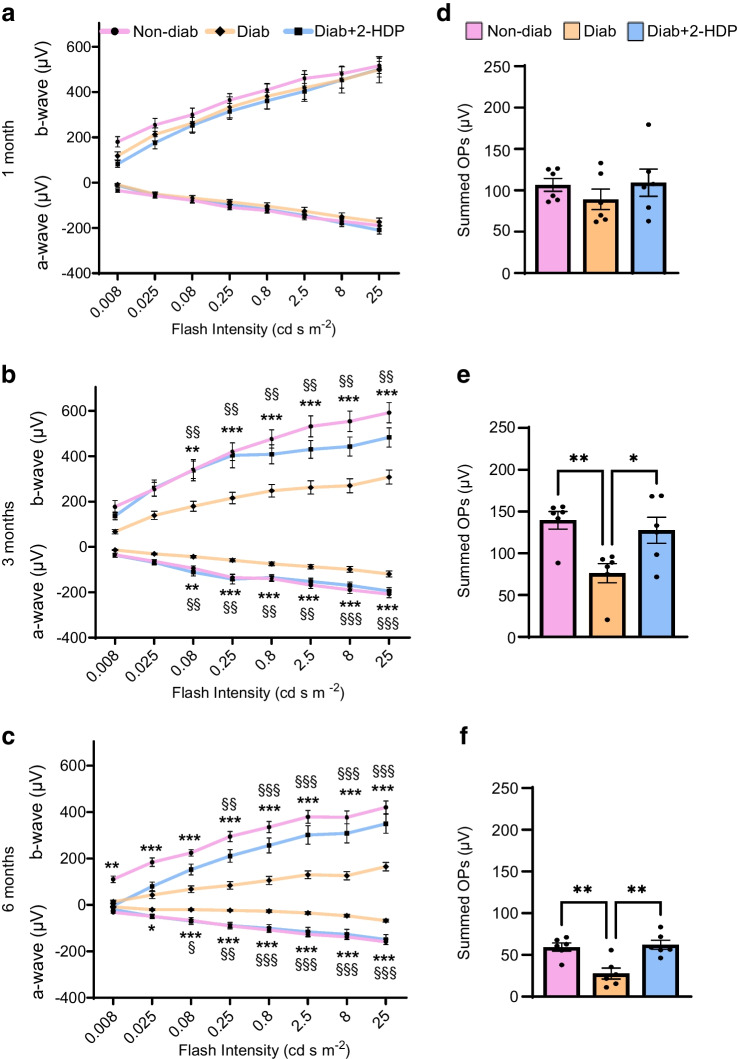
Deterioration of retinal neurophysiological function is attenuated by 2-HDP treatment in diabetic rats. Scotopic ERGs were performed on non-diabetic, diabetic and 2-HDP-treated diabetic rats at 1, 3 and 6 months of diabetes. (**a**–**c**) Line graphs showing a-wave and b-wave amplitudes at 1 month (**a**), 3 months (**b**) and 6 months (**c**) of diabetes, at different flash intensities. (**d**–**f**) Bar graphs showing summed OP amplitudes at 1 month (**d**), 3 months (**e**) and 6 months (**f**) of diabetes. *n*=6 rats per group. Line graphs: *p<0.05, **p<0.01 and ***p<0.001 (non-diabetic vs diabetic); ^§^*p*<0.05, ^§§^*p*<0.01 and ^§§§^*p*<0.001 (diabetic vs 2-HDP-treated diabetic). Bar graphs: *p<0.05 and **p<0.01 for the indicated comparisons. Diab, diabetic

Previous reports have documented reductions in ERG a-wave and b-wave amplitudes, as well as summed OP amplitudes, in both diabetic individuals and rodent models of diabetes [33]. We evaluated the long-term effects of 2-HDP on diabetes-induced changes in neuroretinal function by analysing scotopic flash ERG responses at 1, 3 and 6 months after diabetes induction in rats. No differences in ERG a-wave, b-wave or summed OP amplitudes were observed when comparing non-diabetic rats with diabetic rats after 1 month of diabetes (Fig. 2a, d). However, all components of the ERG waveforms were significantly diminished in diabetic rats after 3 months of diabetes and this worsened by the 6 month time point (Fig. 2b, c, e, f). Impaired ERG responses at 3 months and 6 months were attenuated by treatment with 2-HDP, suggesting that this drug is effective in preserving neuroretinal function in diabetic rats (Fig. 2b, c, e, f).

### 2-HDP confers protection against synaptic alterations and neurodegeneration

Synaptic dysfunction and neurodegeneration have both been implicated in the neurophysiological deficits observed in the diabetic retina [7, 34]. Therefore, we sought to determine whether 2-HDP could provide protection against synaptic and neurodegenerative changes in the retinas of diabetic rats.

#### 2-HDP preserves synaptic integrity

**Fig. 3:**
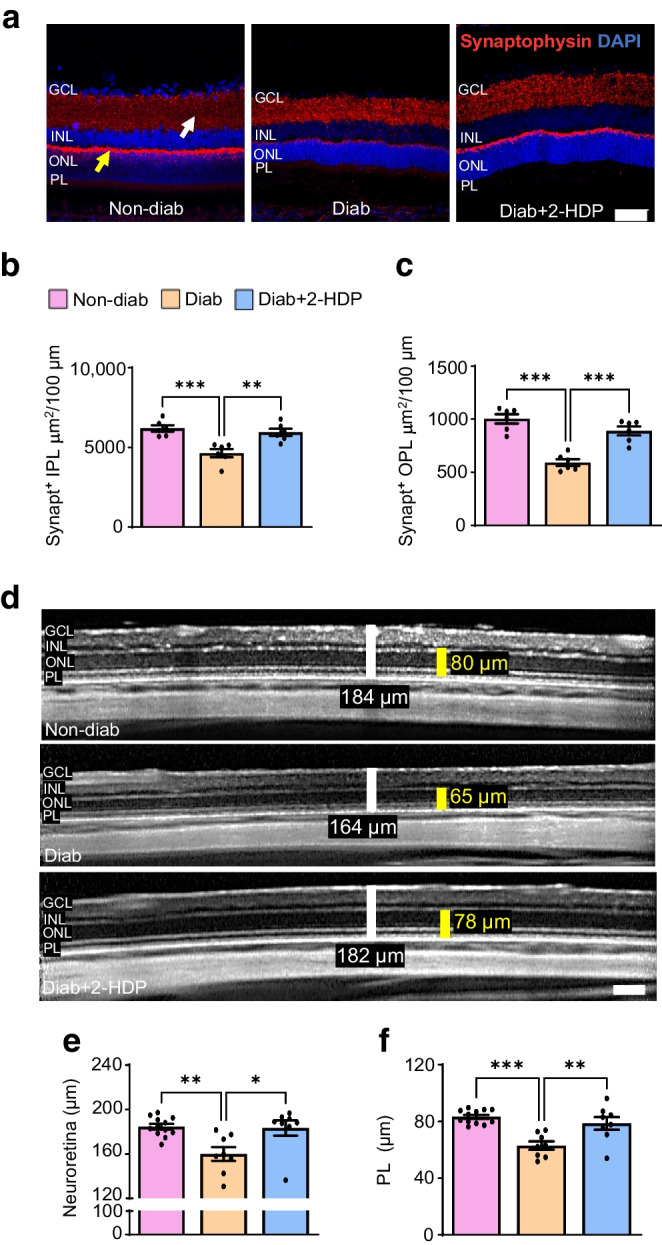
Synaptic and neurodegenerative changes are prevented by 2-HDP treatment in diabetic rats. (**a**) Representative retinal cryosection images from each experimental group after 6 months of diabetes, stained for synaptophysin (red) and DAPI (blue, nuclei). Synaptophysin-positive areas in the inner plexiform layer and outer plexiform layer are marked by white and yellow arrows, respectively. Scale bar, 50 μm. (**b**, **c**) Quantification of synaptophysin-positive areas in the inner (**b**) and outer (**c**) plexiform layer, normalised per 100 μm of retinal length. *n*=6 rats per group. (**d**) Representative SD-OCT images at 3 months of diabetes showing retinal (white) and photoreceptor layer (yellow) thickness measurements. Scale bar, 100 μm. (**e**, **f**) Bar graphs of retinal (**e**) and photoreceptor layer (**f**) thicknesses at 3 months. *n*=8–12 rats per group. *p<0.05, **p<0.01, ***p<0.001, for the indicated comparisons. Diab, diabetic; GCL, ganglion cell layer; IPL, inner plexiform layer; OPL, outer plexiform layer; PL, photoreceptor layer; Synapt, synaptophysin

Synaptophysin, a synaptic vesicle protein, serves as a measurable indicator for functional presynaptic terminals in the retina and plays a vital role in various presynaptic functions, including neurotransmitter release [35]. In diabetes, there is a reduction in synaptophysin levels in the retinas of diabetic rats and in post-mortem retinas from humans; this is believed to disrupt retinal neurotransmission and thus visual pathway function [36, 37]. In this study, synaptophysin-positive areas in retinal cryosections were visualised using confocal microscopy (Fig. 3a). Consistent with previous findings, diabetes led to a decrease in synaptophysin-positive areas in both the inner and outer plexiform layers compared with non-diabetic retinas (Fig. 3a–c). Treatment with 2-HDP protected against alterations in the expression of this protein (Fig. 3a–c).

#### 2-HDP prevents diabetes-induced retinal thinning

SD-OCT imaging has been extensively used to non-invasively examine neurodegeneration in both diabetic individuals and experimental diabetes models [38, 39]. These studies have revealed a progressive thinning of the retina, which may occur before the detectable microvascular pathology characteristic of DRD [40]. We evaluated the protective effects of 2-HDP against diabetes-induced retinal and photoreceptor layer thinning by analysing SD-OCT images taken 1 and 3 months after diabetes induction in STZ-induced diabetic rats. At 1 month, there were no differences in retinal or photoreceptor layer thicknesses among non-diabetic, diabetic and 2-HDP-treated diabetic rats (ESM Fig. 1). However, after 3 months of diabetes, retinal and photoreceptor layer thinning was evident in diabetic rats; this thinning was prevented by 2-HDP treatment (Fig. 3d–f).

#### 2-HDP protects against neuronal cell loss in the diabetic retina

**Fig. 4:**
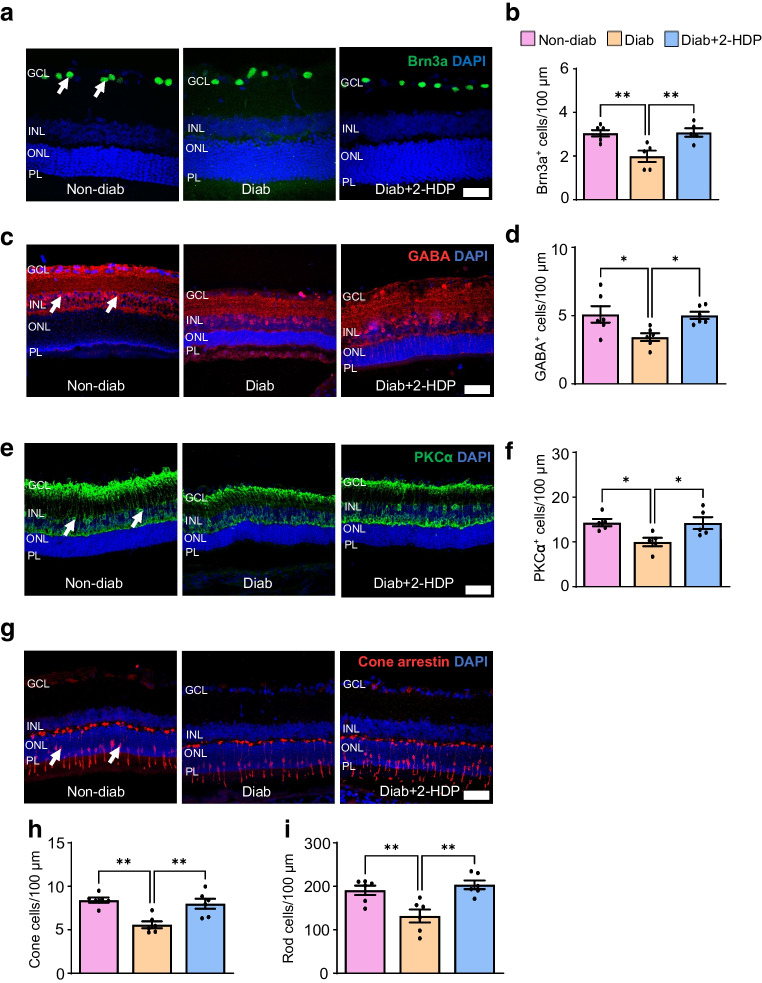
2-HDP prevents the loss of retinal neurons after 6 months of diabetes duration in rats. (**a**) Representative retinal cryosections from each experimental group stained for brain-specific homeobox protein 3a (Brn3a)-positive RGCs (green, arrows) and DAPI (blue, nuclei). (**b**) Bar graph showing numbers of Brn3a-positive RGCs in the ganglion cell layer, normalised to 100 μm retinal length. (**c**) Representative cryosections stained for GABAergic amacrine cells (red, arrows) and DAPI. (**d**) Bar graph showing numbers of GABAergic amacrine cells in the INL, normalised to 100 μm retinal length. (**e**) Representative cryosections stained for protein kinase Cα (PKCα)-positive bipolar cells (green, arrows) and DAPI. (**f**) Bar graph showing numbers of PKCα-positive bipolar cells in the INL, normalised to 100 μm retinal length. (**g**) Representative cryosections stained for arrestin-positive cones (red, arrows) and DAPI. (**h**, **i**) Bar graphs showing the numbers of cone and rod photoreceptors in the photoreceptor layer, normalised to 100 μm retinal length. *n*=5 or 6 rats per experimental group. Scale bar, 50 μm. *p<0.05, **p<0.01. Brn3a, brain-specific homeobox protein 3a; Diab, diabetic; GABA, γ-aminobutyric acid; GCL, ganglion cell layer; PKCα protein kinase Cα; PL, photoreceptor layer

In addition to photoreceptor layer thinning, diabetes-induced retinal neurodegeneration involves the depletion of other neuronal cell types, including RGCs [41], amacrine cells [42] and bipolar cells [43]. To identify which neuronal cell types are protected by 2-HDP in the diabetic retina, we quantified these cells in immunolabelled retinal cryosections from each experimental group of rats after 6 months of diabetes. As shown in Fig. 4a–f, 2-HDP treatment prevented the diabetes-induced loss of RGCs positive for brain-specific homeobox protein 3a, amacrine cells positive for γ-aminobutyric acid (GABAergic cells) and bipolar cells positive for protein kinase Cα. We also examined the effects of 2-HDP on cone and rod photoreceptor degeneration. Cone cells were assessed by staining retinas for cone arrestin, while the number of rod cells was determined by counting DAPI-positive, cone-arrestin-negative nuclei in the outer nuclear layer (ONL). Diabetes caused a decrease in the number of both cone and rod cells compared with non-diabetic controls, and this decrease was prevented by the administration of 2-HDP (Fig. 4g–i).

### Impact of 2-HDP on vascular alterations in the diabetic retina

Our data thus far suggest that 2-HDP exerts a strong neuroprotective effect in the retina of diabetic rats. Consequently, we sought to investigate whether it also exhibits protective effects against the characteristic vascular changes associated with DRD.

#### 2-HDP prevents retinal arteriolar dilation

**Fig. 5:**
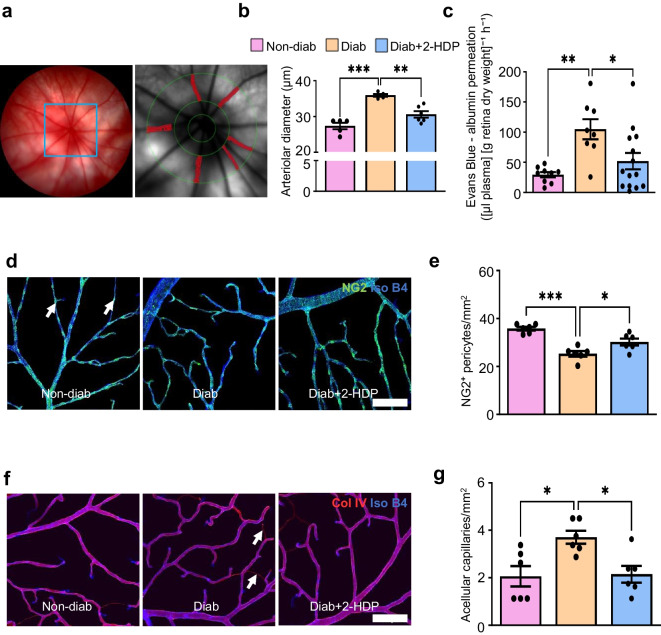
2-HDP prevents retinal vascular pathophysiology and pathology in diabetic rats. (**a**) Representative retinal fundus image with an inset showing an ARIA-processed image of arteriole diameters (red) measured 1–2 disc diameters from the optic nerve head. (**b**) Bar graph showing that 2-HDP prevents retinal arteriolar vasodilation after 3 months of diabetes. *n*=5 or 6 rats/group. (**c**) Bar graph of mean retinal Evans Blue leakage showing reduced vascular permeability with 2-HDP after 3 months of diabetes. *n*=8–14 rats/group. (**d**) Representative retinal wholemounts immunolabelled for neural/glial antigen 2-positive pericytes (green, arrows) and isolectin B4 (pseudo-coloured blue, endothelial cells) after 6 months of diabetes. Scale bar, 100 μm. (**e**) Bar graph quantifying capillary pericytes normalised per mm^2^ of retina. *n*=6 rats/group. (**f**) Representative retinal wholemounts immunostained for Col IV (red) and co-labelled with isolectin B4 (pseudo-coloured blue) 6 months after diabetes induction. Arrows indicate Col IV-positive, isolectin B4-negative acellular capillaries. Scale bar, 100 μm. (**g**) Bar graph quantifying acellular capillaries, normalised per mm^2^ of retina. *n*=6 rats/group. *p<0.05, **p<0.01, ***p<0.001. Diab, diabetic; Iso B4, isolectin B4; NG2, neural/glial antigen 2

The impairment of retinal blood flow autoregulation is one of the earliest vascular changes detected in the diabetic retina [44]. Recently, we have established a connection between this impairment and the progression of both the vascular and neuropathological lesions observed in DRD [25]. Retinal arteriolar dilation serves as an indicator for the loss of retinal blood flow autoregulation [45]. Thus, we employed colour fundus imaging in conjunction with ARIA vessel analysis software to investigate the effects of 2-HDP on this retinal vascular alteration after 3 months of diabetes in rats (Fig. 5a). As expected, mean retinal arteriolar diameters were increased in the untreated diabetic group compared with the non-diabetic control group (Fig. 5b). Treatment with 2-HDP effectively prevented this increase (Fig. 5b).

#### 2-HDP reduces retinal vascular hyperpermeability

Vascular hyperpermeability plays a significant role in causing vision loss in DRD [46]. To evaluate the impact of diabetes on retinal vascular permeability in the absence and presence of 2-HDP, the Evans Blue dye technique was employed. Evans Blue extravasation was markedly increased in the retinas of diabetic rats after 3 months of diabetes, an effect that was largely abrogated in the 2-HDP-treated rats (Fig. 5c).

#### 2-HDP protects against microvascular damage

The microvascular damage observed during the advanced stages of DRD, characterised by pericyte loss and the formation of acellular capillaries, is considered the primary driver of ischaemia-induced neovascularisation in this disease [4]. We evaluated the presence of pericytes in retinal whole mounts from each experimental group of rats by immunolabelling for the pericyte marker neural/glial antigen 2 proteoglycan (Fig. 5d). Our findings revealed a decrease in pericyte numbers in rats after 6 months of diabetes, with some protection being provided by the administration of 2-HDP (Fig. 5e). Acellular capillaries were visualised and quantified by immunolabelling retinal whole mounts using the endothelial cell marker isolectin B4 together with the basement membrane marker collagen IV (Col IV) (Fig. 5f). Similar to our pericyte results, 2-HDP was found to attenuate the rise in acellular capillary formation in the retinas of diabetic rats (Fig. 5g).

#### Effects of 2-HDP on recognised pathways of neurovascular dysfunction and degeneration in the diabetic retina

In DRD, neurovascular dysfunction and neurodegeneration have been linked to proinflammatory signalling and glutamate excitotoxicity [7]. To understand how 2-HDP provides neuro- and vasoprotective effects in the diabetic retina, we investigated its impact on these pathological mechanisms after 3 months of diabetes in rats.

**Fig. 6:**
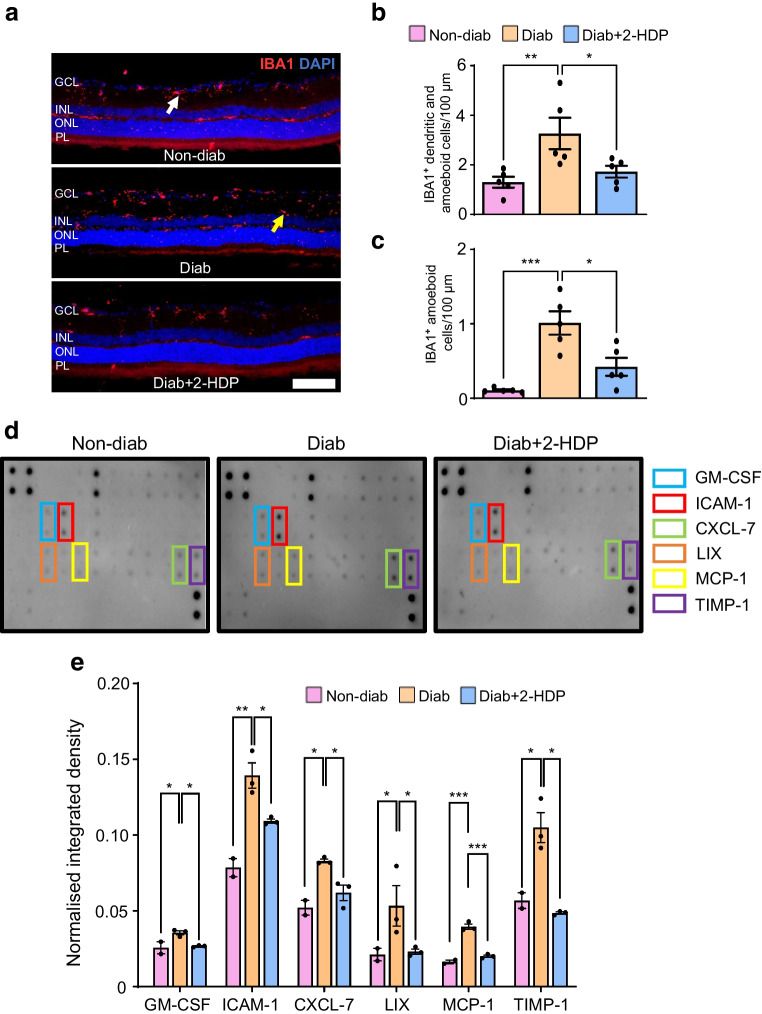
2-HDP prevents microglial and proinflammatory changes in the diabetic retina of rats. (**a**) Representative retinal cryosections stained for ionised calcium binding adaptor molecule 1 (IBA1; red) and counterstained with DAPI (blue) after 6 months of diabetes. Dendritic microglia are marked by white arrows and activated ‘amoeboid’ microglia are marked by yellow arrows, respectively. Scale bar, 50 μm. (**b**, **c**) Bar graphs showing numbers of total (**b**) and activated (**c**) microglia, normalised to 100 μm retinal length. *n*=5 rats per experimental group. (**d**) Representative cytokine array images from non-diabetic, diabetic and 2-HDP-treated diabetic rats after 3 months of diabetes. Each cytokine is spotted in duplicate, with the locations of GM-CSF, ICAM-1, CXCL-7, LIX, MCP-1 and TIMP-1 indicated. (**e**) Quantitative analysis of cytokine arrays was performed by densitometry, with values normalised to positive control spots on each membrane. GM-CSF, ICAM-1, CXCL-7, LIX, MCP-1 and TIMP-1 were upregulated in the diabetic retina and reduced by 2-HDP treatment. *n*=6 rats per experimental group with 2 or 3 technical replicates per group. *p<0.05, **p<0.01, ***p<0.001. Diab, diabetic; IBA1, ionised calcium binding adaptor molecule 1; GCL, ganglion cell layer; PL, photoreceptor layer

We previously demonstrated that 2-HDP reduces proinflammatory responses in Müller glia and decreases the proportion of activated (amoeboid) microglia in the retina after 3 months of diabetes [23]. In this study, we visualised microglia in retinal cryosections using confocal microscopy after 6 months of diabetes (Fig. 6a). Consistent with previous findings, diabetes increased both the total microglial population and the number of activated microglia compared with non-diabetic retinas (Fig. 6b, c). Treatment with 2-HDP reduced both overall microglial numbers and activated microglia in diabetic retinas (Fig. 6a–c). The broader impact of 2-HDP on diabetes-induced changes in retinal inflammatory cytokine expression has not yet been explored. To address this gap, we analysed the retinas of non-diabetic, diabetic and 2-HDP-treated diabetic rats using a rat cytokine antibody array panel. Among the inflammatory factors examined, granulocyte-macrophage colony-stimulating factor (GM-CSF), intercellular adhesion molecule 1 (ICAM-1), CXC motif chemokine ligand 7 (CXCL-7), lipopolysaccharide-induced CXC chemokine (LIX), monocyte chemoattractant protein 1 (MCP-1) and tissue inhibitor of metalloproteinases 1 (TIMP-1) were elevated in the retinas of diabetic rats, with 2-HDP providing partial or full protection against these changes (Fig. 6d, e). Detailed results from the cytokine arrays are presented in ESM Table 5.

**Fig. 7:**
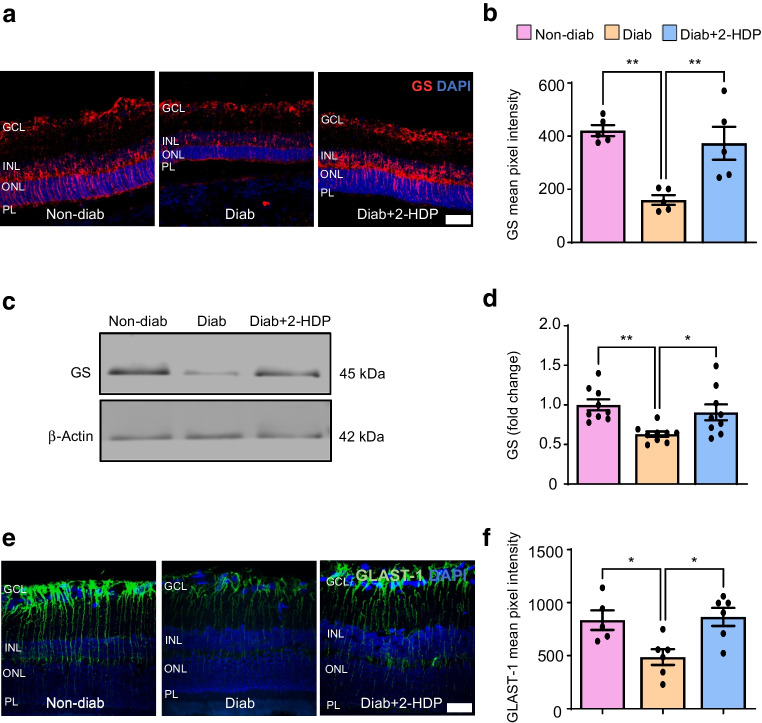
2-HDP improves glutamate-handling mechanisms in the diabetic retina in rats. (**a**) Representative retinal cryosections stained for GS (red) and counterstained with DAPI (blue) after 6 months of diabetes. (**b**) Bar graph showing the mean pixel intensity of GS immunolabelling in the retina. *n*=5 rats per group. (**c**) Representative western blot showing GS protein expression for each experimental group, with each lane representing an individual rat. (**d**) Bar graph showing that 2-HDP prevents GS downregulation after 3 months of diabetes. *n*=9 rats per group. (**e**) Representative retinal cryosections stained for GLAST-1 (green) with DAPI counterstain (blue) after 6 months of diabetes. (**f**) Bar graph showing the mean pixel intensity of GLAST-1 immunolabelling. *n*=5 or 6 rats per group. *p<0.05, **p<0.01. Scale bar, 50 μm. Diab, diabetic; GCL, ganglion cell layer; PL, photoreceptor layer

Müller glia regulate retinal glutamate levels by taking up extracellular glutamate through glutamate aspartate transporter 1 (GLAST-1) and converting it to glutamine via glutamine synthetase. In diabetes, both Müller cell glutamine synthetase (GS) and GLAST-1 are reduced, leading to glutamate accumulation and excitotoxicity [14, 15]. Confocal immunolabelling and western blotting were conducted to assess the impact of 2-HDP on retinal GS protein expression. These studies confirmed diabetes-induced GS downregulation in rats and showed that 2-HDP prevented this effect (Fig. 7a–d). Similar findings were observed when examining the effects of 2-HDP on Müller cell GLAST-1 expression in the diabetic retina (Fig. 7e, f).

### Translational potential of 2-HDP for diabetic retinal disease therapy

Given the promising preclinical effects of 2-HDP on neurovascular pathology in the diabetic rat retina, we aimed to further explore the potential of this drug for treating DRD in humans.

#### FDP-Lys accumulation in the human diabetic retina: implications for translating 2-HDP from animal models to clinical applications

**Fig. 8:**
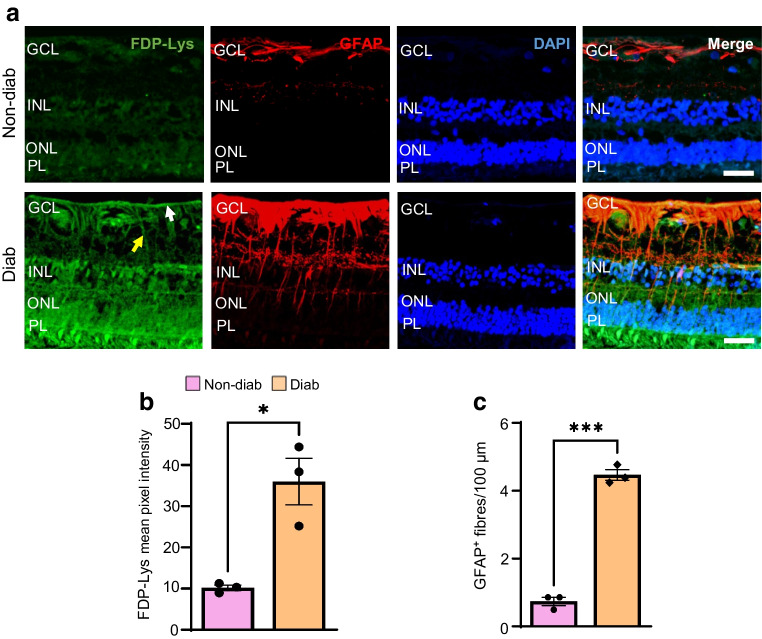
FDP-Lys accumulation in post-mortem retinas from individuals with type 2 diabetes. (**a**) Representative confocal images showing FDP-Lys (green) and GFAP (red) immunoreactivity in retinal sections from a non-diabetic individual and an individual with type 2 diabetes. Nuclei are counterstained with DAPI (blue). In images of retinal sections from individuals with diabetes, white and yellow arrows indicate FDP-Lys immunoreactivity in Müller cell end-feet at the inner limiting membrane and in their radial processes within the inner retina, respectively. (**b**, **c**) Bar graphs quantifying FDP-Lys immunoreactivity (**b**) and the number of GFAP-positive fibres (**c**) in the retinas of non-diabetic individuals and individuals with type 2 diabetes. Data represent three individuals per group. Scale bar, 50 μm. *p<0.05, ***p<0.001. Diab, diabetic; GCL, ganglion cell layer; PL, photoreceptor layer

Previously, we reported that haemoglobin-bound FDP-Lys levels correlate positively with the severity of retinopathy in individuals with type 1 diabetes and individuals with type 2 diabetes [47]. However, it remains unclear whether these adducts accumulate in the human diabetic retina in a manner similar to our observations in diabetic rats [17]. Addressing this question is crucial for assessing the clinical relevance of 2-HDP. To investigate this, we examined human post-mortem retinal sections from non-diabetic individuals and individuals with type 2 diabetes. These sections were co-labelled for FDP-Lys, glial fibrillary acidic protein (GFAP, a marker for Müller glia activation and retinal stress) and the nuclear marker DAPI (Fig. 8a). An increase in FDP-Lys accumulation was observed in retinas from diabetic individuals when compared with their non-diabetic counterparts (Fig. 8a, b). The pattern of FDP-Lys accumulation in the human diabetic retina resembled that previously reported in diabetic rats [17], although the staining was more intense across all layers of the retina, possibly reflecting the longer diabetes duration. Consistent with previous reports, we observed an upregulation of GFAP in human Müller cells during diabetes (Fig. 8a, c) [48], with clear co-localisation of FDP-Lys with Müller cell end-feet at the inner limiting membrane and in the radial processes located in the inner retina (Fig. 8a).

#### Membrane permeability of 2-HDP: implications for preclinical and clinical development

**Fig. 9:**
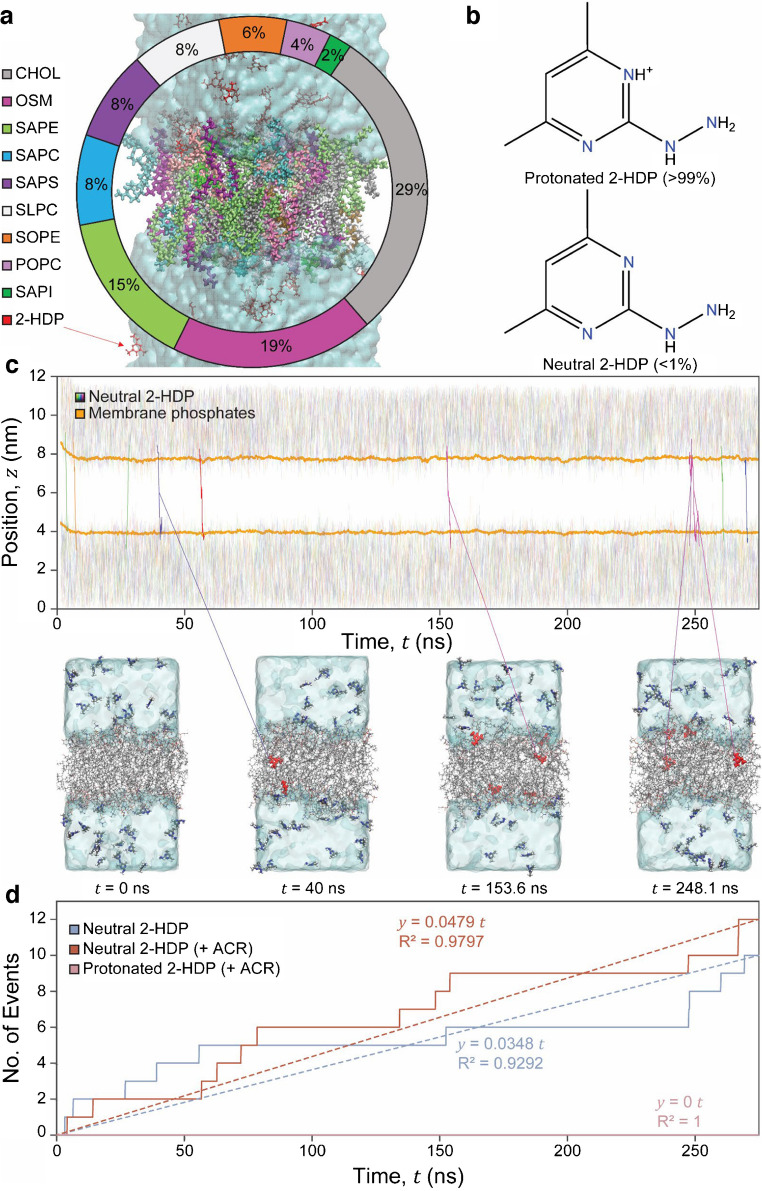
MD simulations of 2-HDP permeability across a human BBB model as a proxy for the iBRB. (**a**) Three-dimensional atomic-resolution representation of a human apical BBB microvascular endothelial cell membrane-mimetic system, used as a proxy to assess 2-HDP permeability across the human iBRB. The lipid composition includes cholesterol (CHOL), *N*-oleoyl-d-erythro-sphingosylphosphorylcholine (OSM), 1-stearoyl-2-arachidonoyl-sn-glycero-3-phosphoethanolamine (SAPE), 1-stearoyl-2-arachidonoyl-sn-glycero-3-phosphocholine (SAPC), 1-stearoyl-2-arachidonoyl-sn-glycero-3-phospho-l-serine (SAPS), 1-stearoyl-2-linoleoyl-sn-glycero-3-phosphocholine (SLPC), 1-stearoyl-2-oleoyl-sn-glycero-3-phosphoethanolamine (SOPE), 1-palmitoyl-2-oleoyl-sn-glycero-3-phosphocholine (POPC) and 1-stearoyl-2-arachidonoyl-sn-glycero-3-phosphoinositol (SAPI). The surrounding solution (150 mmol/l NaCl and water) is depicted as a semi-transparent cyan volume; see ESM Methods for full details on system construction, MD parameters and the theoretical framework and analysis used to calculate permeability. (**b**) Chemical structures of protonated and neutral 2-HDP, with the protonated form (>99%) dominating under physiological pH conditions. (**c**) Time-resolved centre-of-mass *z* positions of 40 neutral 2-HDP molecules (each assigned a unique colour along a rainbow spectrum) relative to membrane phosphate groups (orange) during a 275 ns NPT (constant number of atoms, temperature and pressure)-MD simulation. Representative snapshots at key time points show the initial configuration as well as some instances where neutral 2-HDP was passively translocating across the membrane, with molecules in the transbilayer region highlighted in red. (**d**) Accumulation of 2-HDP translocation events over time, comparing transport rates and linear fits for neutral 2-HDP with (crimson) and without (sky blue) equimolar ACR. Protonated 2-HDP (pink) showed no translocation events even with the leakier membrane in the presence of ACR. Neutral 2-HDP exhibited rapid translocation, which was further enhanced by ACR, yielding permeability within same order of magnitude as caffeine (see ESM Table 6 and ESM Fig. 2). The *R*^2^ values were calculated from linear regression on transition events logged over time, constrained to *y*=0 at *t*=0, representing the accumulated translocation events as a function of simulation time and emphasising overall trends in permeability and transport rate convergence rather than representing fine-grained temporal fluctuations

We previously demonstrated that 2-HDP reaches the retinas of diabetic rats when administered through their drinking water at concentrations equivalent to those used in this study [23]. To assess its potential for systemic delivery in humans, we used MD simulations to investigate its passive permeability properties. An existing atomic-level membrane model of the human BBB was employed for these simulations (Fig. 9a) [30, 31], as insufficient lipidomic data presently limits precise modelling of the iBRB. Cheminformatics microspecies abundance analysis revealed that at near-physiological pH, >99% of 2-HDP exists in a protonated form, with <1% in a neutral form (Fig. 9b). MD simulations focused on the permeation of both forms, revealing that the neutral form readily crosses cell membranes, while the protonated form does not (Fig. 9c, d). Furthermore, the presence of ACR in the system was found to enhance the permeation of the neutral form of 2-HDP, while the protonated form remained impermeable (Fig. 9d). To contextualise these results, a benchmark simulation was conducted using caffeine, a well characterised, neutral molecule known to cross the BBB via passive diffusion [49]. The neutral form of 2-HDP exhibited similar permeability to caffeine, supporting its classification as a relatively permeable solute (see Fig. 9d and ESM Table 6, ESM Fig. 2). These findings offer valuable insights into the diffusional properties of 2-HDP across cell membranes, with implications for its clinical translation.

## Discussion

This study demonstrates that the novel ACR scavenger 2-HDP effectively protects against retinal NVU dysfunction in the STZ rat model of diabetes, independent of glucose levels. Specifically, 2-HDP reduced FDP-Lys accumulation, preserved neuroretinal function and prevented synaptic alterations and neuronal cell loss. Furthermore, 2-HDP prevented key vascular changes associated with DRD, including arteriolar dilation, vascular hyperpermeability and capillary dropout. Our findings of FDP-Lys accumulation in human diabetic retinas, along with in silico analyses of 2-HDP’s permeability properties also suggest promising translational potential for this compound within the context of human DRD.

Over the past 25 years, the role of the NVU in the pathogenesis of DRD has been increasingly recognised, leading to its reclassification as a neurovascular rather than just a microvascular disease of the retina [50]. Various studies have highlighted dysfunctional changes in the retinal NVU during the onset and progression of DRD, including disrupted neurovascular coupling, retinal neurodegeneration, impaired glial cell function, neuroinflammation and microvascular abnormalities [4, 7, 33]. Despite these findings, the exact biochemical mechanisms driving NVU dysfunction in DRD have remained unclear. Our findings, using 2-HDP, suggest that ACR and the subsequent accumulation of FDP-Lys adducts within cells of the retinal neuropile make a significant contribution to retinal NVU dysfunction in diabetes.

The results of this study, together with our earlier work, provide important mechanistic insights into how ACR and FDP-Lys contribute to NVU dysfunction in the diabetic retina. We previously identified that FDP-Lys adducts initially accumulate in Müller glia during experimental diabetes, later spreading to neurons in the RGC layer and INL [17]. We also demonstrated that FDP-Lys accumulation in Müller glia triggers oxidative stress and proinflammatory signalling, contributing to retinal microglial activation [23]. In this study, we further analysed inflammatory factors linked to ACR formation and FDP-Lys accumulation in the diabetic retina, identifying the upregulation of GM-CSF, ICAM-1, CXCL-7, LIX, MCP-1 and TIMP1, all of which were reduced by 2-HDP treatment. Each of these factors is known to play a key role in processes relevant to the pathology of DRD, including microglial activation, immune cell recruitment, leukostasis, iBRB breakdown, capillary occlusion, neovascularisation and neurodegeneration [51–58]. These findings could help to explain how ACR and FDP-Lys formation in Müller glia drives broader NVU dysfunction in the diabetic retina. In addition, our data indicate that beyond inflammatory signalling, ACR and FDP-Lys accumulation may contribute to neurodegeneration by disrupting Müller glia glutamate handling. Notably, 2-HDP treatment prevented the diabetes-induced downregulation of GLAST-1 and GS, two key proteins for which dysfunction is believed to drive extracellular glutamate accumulation and neurotoxicity in the diabetic retina [14, 15]. Finally, the protective effects of 2-HDP on neurodegeneration in the diabetic retina may be attributed not only to improved Müller glia function and reduced proinflammatory signalling but also to the direct scavenging of ACR and the prevention of FDP-Lys accumulation in retinal neurons. Elevated ACR levels are directly neurotoxic to various types of brain neurons [18, 59, 60], and this mechanism has been implicated in several neurodegenerative disorders, including Alzheimer’s disease, Parkinson’s disease and multiple sclerosis [61].

While our understanding of DRD as a neurovascular disease has grown substantially in recent years, the contribution of retinal gliotic and neurodegenerative changes to the development of the sight-threatening microvascular complications of this disease has remained largely unclear. Our current and previous data shed light on this issue, demonstrating that ACR and FDP-Lys predominantly accumulate in Müller glia and neurons of the diabetic rat retina [17], yet 2-HDP effectively prevents not only Müller glia [23] and neuronal damage but also microvascular pathophysiology and pathology. These findings suggest that gliotic and neurodegenerative changes significantly contribute to the microvascular complications observed in DRD. Such observations underscore the necessity for the development and clinical testing of NVU-targeted therapeutics for DRD, which could provide a more comprehensive approach to its management by addressing all facets of the disease and preventing progression to sight-threatening microvascular stages. Interestingly, the concept of NVU-targeted therapies is also gaining momentum in neurodegenerative diseases of the brain, reflecting a shift in focus from solely targeting neuronal cells to sustaining NVU function to promote neuronal survival rate [62].

The findings from this study suggest that 2-HDP holds considerable promise as an NVU-targeted therapeutic agent for clinical translation in DRD. While increased FDP-Lys accumulation has previously been documented in fibrovascular tissues from individuals with PDR [63], this study is the first to demonstrate that FDP-Lys also accumulates in the human retinal neuropile during diabetes, aligning with our earlier observations in diabetic rats [17, 23]. This discovery suggests the likely involvement of ACR and FDP-Lys in the pathogenesis of human DRD and highlights the therapeutic potential of ACR scavengers, such as 2-HDP, in treating this condition.

Our MD simulations suggest that the neutral form of 2-HDP can readily permeate the BBB, which shares structural similarities with the iBRB. Although the neutral form accounts for only ~1% of 2-HDP at physiological pH, its overall permeability is likely greater than expected from the diffusion of the neutral species alone. In vivo, the permeability of small molecules across biological membranes is influenced by protonation–deprotonation dynamics, which depend on the local microenvironment, pH and the relative rates of ionisation and permeation [64, 65]. Additionally, positively charged molecules can destabilise the lipid bilayer, further enhancing passive diffusion [66]. While these mechanisms were beyond the scope of our MD simulations, they suggest that 2-HDP likely exhibits significant in vivo permeability, similar to that of clinically used drugs such as amantadine, which shares comparable charge and permeability properties [67]. Considering that over 98% of small-molecule drugs are unable to cross the BBB [68], the demonstrated permeability of 2-HDP highlights its potential as a promising candidate for clinical translation in individuals with diabetes.

Although 2-HDP shows significant potential for clinical application in DRD, several important considerations must be addressed before progressing to human studies. Hydrazine-containing compounds, such as 2-HDP, are relatively uncommon in pharmaceuticals due to their reactivity and potential toxicity [69]. For example, systemic administration of hydrazine-derived compounds such as iproniazid, chlorozotocin and aminoguanidine either failed in clinical trials or were discontinued due to liver or kidney toxicity [69–71]. Another concern regarding the long-term systemic use of ACR scavengers is the risk of forming drug–ACR–protein complexes, which could trigger autoimmune responses by acting as novel antigens to immune cells [72]. While no overt adverse effects of 2-HDP were observed in the rats within this study, comprehensive preclinical safety and toxicity evaluations will be required to support its translational development. In addition, it is important to acknowledge that the 40 mg/kg dose of 2-HDP used in this study is relatively high. This dose was selected based on prior in vivo efficacy studies where it provided significant Müller cell protection without evident toxicity [23]. However, to better define the therapeutic window and determine the minimum effective concentration, both in vitro and in vivo dose–response studies represent a crucial next step. These investigations are essential for guiding future preclinical development and optimising the clinical translation of 2-HDP.

As a newly identified ACR scavenger, further investigation into this compound’s ability to neutralise other lipid peroxidation products and reactive carbonyl species generated during lipid peroxidation and glycation reactions would also be valuable for evaluating its clinical potential and identifying any additional mechanisms of action. While ACR and FDP-Lys are the only lipid peroxidation and lipoxidation products we have previously identified as accumulating in the STZ rat model of diabetes [17], we cannot entirely exclude the possibility that some of the drug’s beneficial effects observed in this study may involve additional effects on reactive carbonyl species and AGE formation. However, previous studies suggest that the closely related compound hydralazine does not directly scavenge glyoxal, a product of lipoxidation or glycation reactions [73]. Nonetheless, if 2-HDP is found to influence a broader range of lipoxidative and carbonyl stress pathways, this could be advantageous, as various lipoxidation and glycation products are known to accumulate in the human diabetic retina with prolonged disease progression [74].

In conclusion, this study shows that 2-HDP provides significant protection against retinal NVU dysfunction associated with diabetes. Our findings not only emphasise the therapeutic potential of ACR detoxification strategies in DRD but also highlight the necessity for further preclinical and clinical investigations into the efficacy of 2-HDP as a treatment. Future research should aim to optimise delivery methods and assess the long-term effects of 2-HDP to ensure both patient safety and treatment effectiveness.

## Supplementary Information

Below is the link to the electronic supplementary material.

ESM (PDF 772 KB)

## Data Availability

The data supporting the findings of this study are available from the corresponding author upon reasonable request.

## Abbreviations

ACR: Acrolein
ARIA: Automated Retinal Image Analyser
BBB: Blood–brain barrier
Col IV: Collagen IV
CXCL-7: CXC motif chemokine ligand 7
DRD: Diabetic retinal disease
ERG: Electroretinogram
FDP-Lys: *N*ε-(3-formyl-3,4-dehydropiperidino)lysine
GFAP: Glial fibrillary acidic protein
GLAST-1: Glutamate aspartate transporter 1
GM-CSF: Granulocyte-macrophage colony-stimulating factor
GS: Glutamine synthetase
2-HDP: 2-Hydrazino-4,6-dimethylpyrimidine
iBRB: Inner blood–retina barrier
ICAM-1: Intercellular adhesion molecule 1
IHC: Immunohistochemistry
INL: Inner nuclear layer
LIX: Lipopolysaccharide-induced CXC chemokine
MCP-1: Monocyte chemoattractant protein 1
MD: Molecular dynamics
NVU: Neurovascular unit
ONL: Outer nuclear layer
OP: Oscillatory potential
PDR: Proliferative diabetic retinopathy
RGC: Retinal ganglion cell
ROI: Region of interest
SD-OCT: Spectral-domain optical coherence tomography
STZ: Streptozocin
TIMP-1: Tissue inhibitor of metalloproteinases 1
VEGF: Vascular endothelial growth factor
