# Supplementary material for: Scavenging acrolein with 2-HDP preserves neurovascular integrity in a rat model of diabetic retinal disease
Source: Diabetologia. 2025 Aug 15;68(11):2609–29. doi: 10.1007/s00125-025-06515-2 (PMC12534245; doi:10.1007/s00125-025-06515-2)
Supplement: Supplementary file 1 — ESM (PDF 772 KB) [file 125_2025_6515_MOESM1_ESM.pdf]

Scavenging acrolein with 2-HDP preserves neurovascular integrity in a rat model of diabetic retinal disease

ESM Methods

Computational chemistry

Molecular dynamics simulations

The permeation of 2-HDP was investigated by atomic-resolution equilibrium molecular dynamics (MD) simulations constructed and performed using GROMACS (version 2021.2) [1], under the CHARMM36 additive empirical potential with explicit transferable intermolecular potential with 3 points (TIP3P) water [2-4]. Four simulations, each lasting 275 ns, were conducted to assess passive transbilayer diffusion under different solute conditions: (1) 40× neutral 2HDP, (2) 40× neutral 2HDP + 40× acrolein, (3) 40× protonated 2HDP, and (4) 40× protonated 2HDP + 40× acrolein. A separate caffeine benchmark simulation was performed under similar conditions to facilitate comparison with the 2-HDP results. Neutral and protonated 2-HDP physicochemical parameters including partial charges and bond strengths were obtained using the CGenFF force field parameterisation method [5-7].

System building and lipid model

The lipid bilayer system was constructed to simulate the human blood-brain barrier (BBB) as a surrogate for the inner blood-retinal barrier (iBRB). This choice was made due to the lack of sufficient detailed lipidomic data for the iBRB, including the necessary head and tail group compositions. The BBB lipid bilayer model included 96 lipids with a composition reflective of a typical human microvascular BBB endothelial apical membrane: a mixture of sphingomyelin, cholesterol, and various glycerophospholipids [8]. While not representing the exact lipidomic profile of the iBRB, this system served as a reasonable approximation based on shared structural and functional characteristics of the barriers. Solute molecules were randomly inserted into the bilayer system using the `gmx insert-molecules` tool, replacing water molecules, and the system was equilibrated prior to production simulations.

The table below outlines the lipid composition of the BBB membrane model:

Lipid (abbreviation)	Count (#)
N-oleoyl-d-erythro-sphingosylphosphorylcholine (OSM)	18
Cholesterol (CHOL)	28
1-palmitoyl-2-oleoyl-sn-glycero-3-phosphocholine (POPC)	4
1-stearoyl-2-arachidonoyl-sn-glycero-3-phosphocholine (SAPC)	8
1-stearoyl-2-arachidonoyl-sn-glycero-3-phosphoethanolamine (SAPE)	14
1-stearoyl-2-oleoyl-sn-glycero-3-phosphoethanolamine (SOPE)	6
1-stearoyl-2-arachidonoyl-sn-glycero-3-phospho-L-serine (SAPS)	8
1-stearoyl-2-linoleoyl-sn-glycero-3-phosphocholine (SLPC)	8
1-stearoyl-2-arachidonoyl-sn-glycero-3-phosphoinositol (SAPI)	2

Simulation setup

All simulations were performed in the NPT ensemble (i.e., constant number of particles, pressure, and temperature) with water and ions, lipids, and monitored species (i.e., acrolein and 2HDP molecules) coupled separately to heat baths of temperature $T = 440$ K (~167°C) with time constant $\tau_T = 0.5$ ps using the Bussi−Donadio−Parrinello 'v-rescale' thermostat method [9]. Ensemble equilibrium pressure (P=1.0 bar) within the 3D periodic simulation box was enforced by weak semi-isotropic coupling using the 'berendsen' barostat algorithm [10] applied with time constant $\tau_P = 5$ ps and compressibilities $\kappa_Z = \kappa_{XY} = 4.6 \times 10^{-5}$ bar^{-1}. Mean simulation box volumes were ~5.7×5.7×11.4 nm^3, yielding mean system per solute concentrations of ~180 mM. Solute molecules were introduced into a solvated lipid bilayer system with a neutralising concentration of Na^+/Cl^- ions at 150 mM to avoid potential Ewald summation-derived artifacts [11, 12]. Simulations used a timestep of 2 fs, with hydrogen-containing bonds constrained using LINear Constraint Solver (LINCS) [13]. Non-bonded interactions were calculated with a cutoff of 1.0 nm for Lennard-Jones and short-range Coulombic interactions, and long-range electrostatics were computed using Particle Mesh

Ewald summation [14, 15]. Simulation trajectories were saved every 5 picoseconds to capture detailed translocation events and bilayer interactions.

Permeability and rate calculations

Two general approaches exist for calculating permeability in MD simulations: Fick's law [16] translocation counting-based methods and inhomogeneous solubility-diffusion (ISD) methods [17, 18]. This study employed a translocation counting-based approach to directly observe and quantify solute translocation events as the authors have done previously [19-21]. To recapitulate, a translocation event was defined as a solute molecule crossing from one bulk solution region to the opposite bulk region, passing through a plane 1.0 nm beyond the bilayer's mean positions of the phosphate head groups, demarking the bilayer interface (see Figure 9C). The rate constant, k, representing the frequency of translocation events, was determined by calculating the cumulative number of events over time. A steady-state value for k, was defined where its derivative, $\frac{dk}{dt}$, dropped below 0.004 events/ns. The permeability coefficient, P, was then computed as:

$$P = \frac{J}{A} = \frac{1}{2} \times \frac{r}{AC}$$

where r is the steady-state rate of events per solute molecule (i.e., $r = \frac{k}{N}$), A is the lateral (i.e., x-y) cross-sectional area of the bilayer obtained from the dimensions of the periodic simulation box, and C is the solute concentration, $C = \frac{N}{V}$. The factor of 2 accounts for the bidirectional flux observed in MD simulations compared to the monodirectional flux in experiments (e.g., transwell assays). Elevated temperatures allowed for efficient sampling, with each rate constant converging to steady-state values within ~50 ns.

To extrapolate high-temperature simulations to physiologically relevant conditions, a regression model linking simulated and experimental permeabilities was employed, based on a dataset of 18 diverse permeating compounds spanning several orders of magnitude (~10^{-7} to ~10^{-2} cm s^{-1}) [16]. This model enables comparison of arbitrary solute compounds to the

pre-existing, benchmarked dataset, with a reported mean error of 0.44–0.55 orders of magnitude compared to experimental data. A single simulation per compound at 440 K allowed us to obtain extrapolated permeabilities at 37°C.

Trajectory data were analysed using MDAnalysis v2.8.0, a Python-based library for molecular simulation analysis [22]. MDAnalysis identified translocation events, computed translocation rates, and derived spatiotemporal distributions of solutes across the bilayer. Custom Python scripts built with MDAnalysis automated the identification of solute positions and translocation events, providing time series data for k, r, A, V, C, and P.

Comparison to caffeine

The permeability of neutral 2-HDP is of the same order of magnitude as caffeine, as indicated by the translocation time series data presented in ESM Figure 2. For a comparison of calculated equilibrium permeabilities, refer to ESM Table 6.

ESM Tables

ESM Table 1. Demographics of the non-diabetic and diabetic human sample donors.

Donor	Sex	Age	Ophthalmic History
Non-diabetic	Male	68 years old	Cataract
Non-diabetic	Female	61 years old	Unremarkable
Non-diabetic	Female	63 years old	Unremarkable
Type 2 diabetes	Male	77 years old	Cataract Surgery
Type 2 diabetes	Female	73 years old	Cataract Surgery
Type 2 diabetes	Male	69 years old	Unknown

ESM Table 2: Antibodies used in immunohistochemistry of human retinas

Antibody	Dilution	Company	Catalogue no.
FDP-Lys	1:500	JaICA	mAb5F6
GFAP	1:1000	DAKO	Z0334
Donkey anti-Mouse Alexa Fluor 488	1:200	Invitrogen	A21202
Donkey anti-Rabbit Alexa Fluor 568	1:200	Invitrogen	A10042

ESM Table 3. Antibodies used for rat immunohistochemistry. Primary and secondary antibodies listed by name, dilution, manufacturer, and catalogue number.

Antibody	Dilution	Company	Catalogue no.
BRN3A	1:200	Abcam	ab245230
Collagen IV	1:75	Bio-Rad	2150-1470
Cone-Arrestin	1:1000	Merck Millipore	ab15282
GABA	1:500	Sigma-Aldrich	A2052
GLAST-1	1:100	Alomone labs	AGC-021
GS	1:2000	Abcam	ab73593
IBA1	1:200	WAKO	019-19741
NG2	1:100	Merck	MAB5384-I
PKCα	1:300	Abcam	ab11723
Synaptophysin	1:300	Invitrogen	MA5-14532
Donkey anti-Rabbit Alexa Fluor 488	1:300	Invitrogen	A21206
Donkey anti-Rabbit Alexa Fluor 568	1:300	Invitrogen	A10042
Donkey anti-Mouse Alexa Fluor 488	1:300	Invitrogen	A21202

ESM Table 4. Antibodies used for western blotting. Primary and secondary antibodies listed by name, dilution, manufacturer, and catalogue number.

Antibody	Dilution	Company	Catalogue no.
FDP-Lys	1:500	Abcam	ab240918
GS	1:5000	Abcam	ab73593
β-actin	1:5000	Cell Signaling	3700S
Goat anti–mouse HRP antibody	1:5000	Cell Signaling	7076S
IRDye® 800CW Goat anti-Rabbit IgG Secondary Antibody	1:10000	LI-COR Biosciences	925-32211

ESM Table 5. Full results of the cytokine analysis

Inflammatory factor	Diab vs Non-diab<br>Fold change (P value)	Diab+2-HDP vs Non-diab<br>Fold change (P value)	Diab+2-HDP vs Diab<br>Fold change (P value)
Activin A	1.16 (P>0.05)	1.12 (P>0.05)	0.97 (P>0.05)
Agrin	1.07 (P>0.05)	1.05 (P>0.05)	0.98 (P>0.05)
B7-2/CD86	0.96 (P>0.05)	0.92 (P>0.05)	0.96 (P>0.05)
β-NGF	1.22 (P>0.05)	1.21 (P>0.05)	0.99 (P>0.05)
CINC-1	1.13 (P>0.05)	1.14 (P>0.05)	1.01 (P>0.05)
CINC-2α	0.98 (P>0.05)	1.01 (P>0.05)	1.03 (P>0.05)
CINC-3	0.71 (P>0.05)	0.84 (P>0.05)	1.19 (P>0.05)
CNTF	0.73 (P>0.05)	0.60 (P>0.05)	0.82 (P>0.05)
Fas Ligand	2.31 (P>0.05)	1.54 (P>0.05)	0.67 (P>0.05)
Fractalkine	1.15 (P>0.05)	1.31 (P>0.05)	1.14 (P>0.05)
GM-CSF	1.39 (P<0.05)	1.04 (P>0.05)	0.75 (P<0.05)
ICAM-1	1.77 (P<0.01)	1.39 (P<0.05)	0.78 (P<0.05)

IFN-y	2.06 (P>0.05)	0.87 (P>0.05)	0.43 (P>0.05)
IL-1α	1.00 (P>0.05)	0.80 (P>0.05)	0.79 (P>0.05)
IL-1β	1.01 (P>0.05)	1.08 (P>0.05)	1.07 (P>0.05)
IL-1 R6	1.10 (P>0.05)	0.86 (P>0.05)	0.78 (P>0.05)
IL-2	0.91 (P>0.05)	0.70 (P>0.05)	0.77 (P>0.05)
IL-4	0.91 (P>0.05)	0.58 (P>0.05)	0.64 (P>0.05)
IL-6	0.28 (P>0.05)	0.23 (P>0.05)	0.83 (P>0.05)
IL-10	0.34 (P>0.05)	0.38 (P>0.05)	1.10 (P>0.05)
IL-13	1.57 (P>0.05)	0.85 (P>0.05)	0.54 (P>0.05)
Leptin	1.02 (P>0.05)	1.29 (P>0.05)	1.26 (P>0.05)
LIX	1.84 (P<0.05)	1.169 (P>0.05)	0.63 (P<0.05)
L-Selectin	1.85 (P>0.05)	1.28 (P>0.05)	0.69 (P>0.05)
MCP-1	2.24 (P<0.001)	1.23 (P>0.05)	0.55 (P<0.001)
MIP-3α	1.11 (P>0.05)	0.96 (P>0.05)	0.86 (P>0.05)
MMP-8	1.50 (P>0.05)	1.59 (P>0.05)	1.04 (P>0.05)
PDGF-AA	1.10 (P>0.05)	1.12 (P>0.05)	1.02 (P>0.05)
Prolactin R	0.99 (P>0.05)	0.84 (P>0.05)	0.85 (P>0.05)
RAGE	1.03 (P>0.05)	0.94 (P>0.05)	0.91 (P>0.05)
CXCL7	1.59 (P<0.05)	1.19 (P>0.05)	0.75 (P<0.05)
TIMP-1	1.85 (P<0.05)	0.81 (P>0.05)	0.44 (P<0.05)
TNF-α	1.72 (P>0.05)	1.85 (P>0.05)	1.08 (P>0.05)
VEGF	1.49 (P<0.05)	1.27 (P>0.05)	0.86 (P>0.05)

Abbreviations: B7-2/CD86, B7-2 cluster of differentiation 86; β-NGF, beta-nerve growth factor; CINC-1, cytokine-induced neutrophil chemoattractant-1; CINC-2α, cytokine-induced neutrophil chemoattractant-2 alpha; CINC-3, cytokine-induced neutrophil chemoattractant-3; CNTF, ciliary neurotrophic factor; MIP-3α, macrophage inflammatory protein-3 alpha; MMP-

8, matrix metalloproteinase-8; PDGF-AA, platelet-derived growth factor-AA; Prolactin R, prolactin receptor; RAGE, receptor for advanced glycation end-products; VEGF, vascular endothelial growth factor.

ESM Table 6. MD simulations and equilibrium results.

Input parameters include molecule species, temperature (T), and simulation duration (τ). Results include the number of translocation events, per-molecule translocation rates (r), membrane cross-sectional areas (A), simulation permeabilities (P_{sim}, calculated as $P_{\text{sim}} = \frac{r}{2AC}$), and predicted experimental permeabilities (P_{exp}). P_{exp} was estimated using the regression equation from Jorgensen et al. [21]: $\log_{10}(P_{\text{exp}}) = 1.17\,[\log_{10}(P_{\text{sim@440K}})] - 3.73$.

Molecules	T [K]	τ [ns]	Events	r [s^{-1}]	A [nm^2]	C [mM]	P_{sim} [cm/s]	P_{exp} [cm/s]
40 2-HDP (neutral)	440	275	10	9.09×10^5	31.72	181.87	5.29×10^{-2}	5.97×10^{-6}
40 2-HDP (neutral); 40 acrolein	440	275	12	1.09×10^6	31.93	181.25	6.32×10^{-2}	7.36×10^{-6}
40 2-HDP (+)	440	275	0	n/a	36.16	164.58	n/a	n/a
40 2-HDP (+); 40 acrolein	440	275	0	n/a	36.38	164.04	n/a	n/a
40 Caffeine (benchmark)	440	275	12	1.09×10^6	33.14	182.46	6.03×10^{-2}	6.97×10^{-6}

ESM Figures

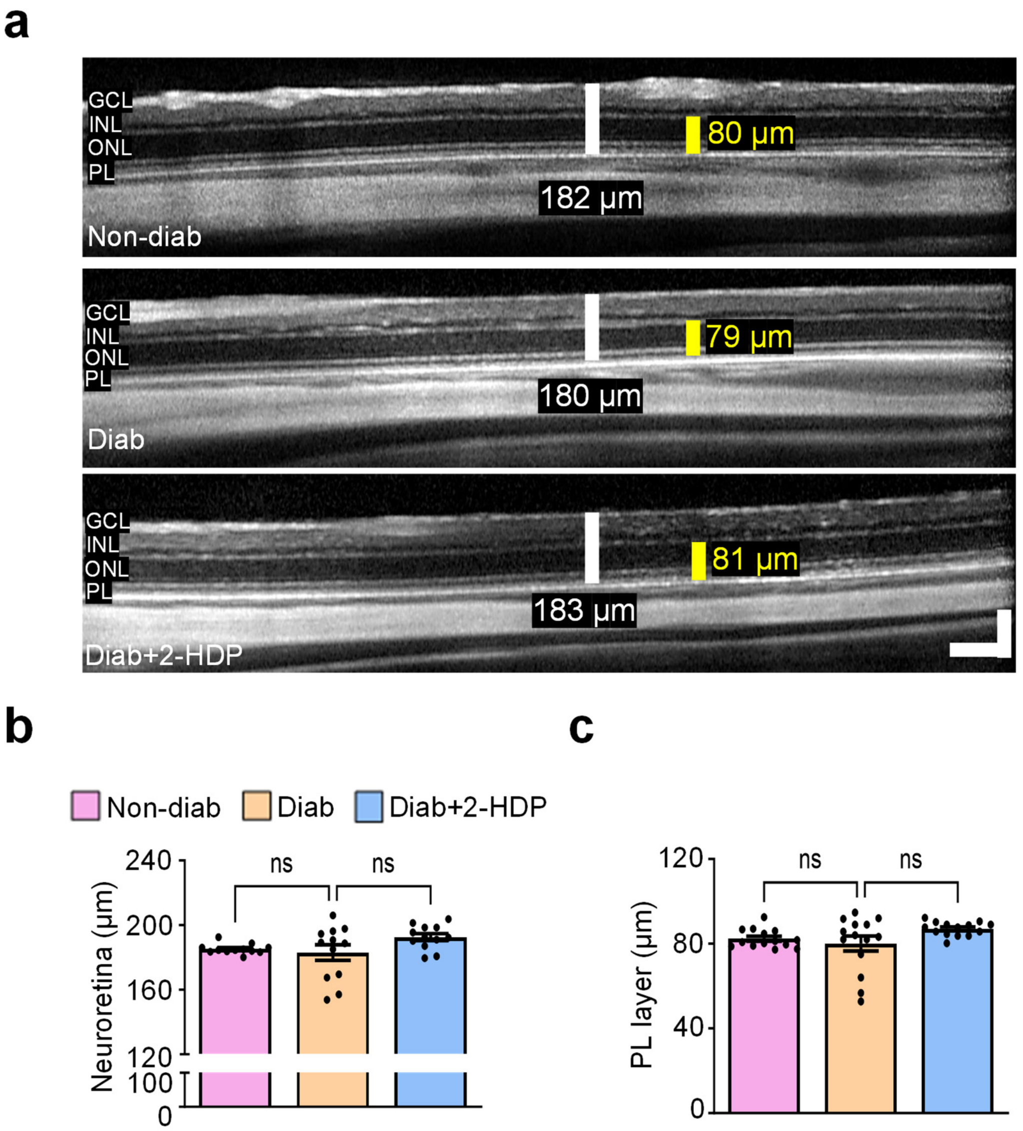

ESM Figure 1. Retinal thickness remains unchanged among non-diabetic, diabetic, and 2-HDP-treated diabetic rats after 1 month of diabetes. (**a**) Representative SD-OCT images of the experimental groups at 1 month of diabetes, showing retinal thickness (white) and photoreceptor layer thickness (yellow). Scale bar: 100 µm. (**b-c**) Bar graphs of retinal and photoreceptor layer thicknesses at 1 month. Data are based on *n*=6 animals per group. Diab, diabetic; GCL, ganglion cell layer; INL, inner nuclear layer; ONL, outer nuclear layer; PL, photoreceptor layer.

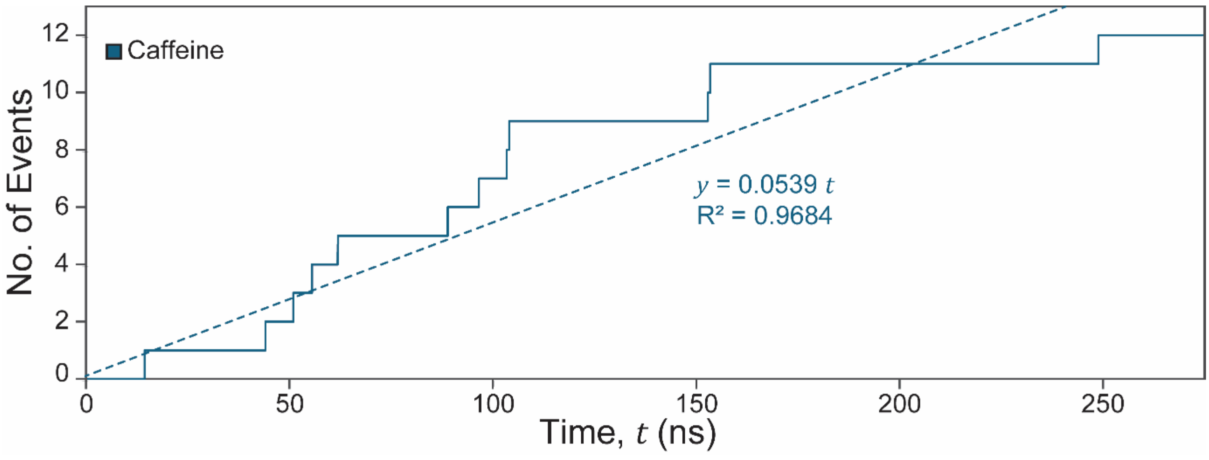

ESM Figure 2. Accumulation of caffeine translocation events over time with linear fit as a benchmark. The R^2 value was calculated from a linear regression of translocation events over time, constrained to y=0 at t=0.

References

[1] Abraham MJ, Murtola T, Schulz R, et al. (2015) GROMACS: High performance molecular simulations through multi-level parallelism from laptops to supercomputers. SoftwareX 1-2: 19-25. https://doi.org/10.1016/j.softx.2015.06.001

[2] Huang J, MacKerell Jr AD (2013) CHARMM36 all-atom additive protein force field: Validation based on comparison to NMR data. Journal of Computational Chemistry 34(25): 2135-2145. https://doi.org/10.1002/jcc.23354

[3] Jorgensen WL, Chandrasekhar J, Madura JD, Impey RW, Klein ML (1983) Comparison of simple potential functions for simulating liquid water. The Journal of Chemical Physics 79: 926-935. https://doi.org/10.1063/1.445869

[4] MacKerell Jr. AD, Bashford D, Bellott M, et al. (1998) All-Atom Empirical Potential for Molecular Modeling and Dynamics Studies of Proteins. The Journal of Physical Chemistry B 102: 3586-3616. https://doi.org/10.1021/jp973084f

[5] Vanommeslaeghe K, Hatcher E, Acharya C, et al. (2010) CHARMM general force field: A force field for drug-like molecules compatible with the CHARMM all-atom additive biological force fields. Journal of Computational Chemistry 31(4): 671-690. https://doi.org/10.1002/jcc.21367

[6] Vanommeslaeghe K, MacKerell Jr. AD (2012) Automation of the CHARMM General Force Field (CGenFF) I: Bond Perception and Atom Typing. Journal of Chemical Information and Modeling 52(12): 3144-3154. https://doi.org/10.1021/ci300363c

[7] Vanommeslaeghe K, Raman EP, MacKerell Jr. AD (2012) Automation of the CHARMM General Force Field (CGenFF) II: Assignment of Bonded Parameters and Partial Atomic Charges. Journal of Chemical Information and Modeling 52(12): 3155-3168. https://doi.org/10.1021/ci3003649

[8] Tewes BJ, Galla HJ (2001) Lipid Polarity in Brain Capillary Endothelial Cells. Endothelium 8(3): 207-220. https://doi.org/10.1080/10623320109051566

[9] Bussi G, Donadio D, Parrinello M (2007) Canonical sampling through velocity rescaling. The Journal of Chemical Physics 126: 014101. https://doi.org/10.1063/1.2408420

[10] Berendsen HJC, Postma JPM, Van Gunsteren WF, Dinola A, Haak JR (1984) Molecular dynamics with coupling to an external bath. The Journal of Chemical Physics 81: 3684-3690. https://doi.org/10.1063/1.448118

[11] Hub JS, De Groot BL, Grubmüller H, Groenhof G (2014) Quantifying artifacts in Ewald simulations of inhomogeneous systems with a net charge. Journal of Chemical Theory and Computation 10: 381-390. https://doi.org/10.1021/ct400626b

[12] Wong-ekkabut J, Karttunen M (2016) The good, the bad and the user in soft matter simulations. Biochimica et Biophysica Acta - Biomembranes 1858(10): 2529-2538. https://doi.org/10.1016/j.bbamem.2016.02.004

[13] Hess B, Bekker H, Berendsen HJC, Fraaije JGEM (1997) LINCS: A Linear Constraint Solver for molecular simulations. Journal of Computational Chemistry 18: 1463-1472. https://doi.org/10.1002/(SICI)1096-987X(199709)18:12<1463::AID-JCC4>3.0.CO;2-H

[14] Ewald PP (1921) Die Berechnung optischer und elektrostatischer Gitterpotentiale. Annalen der Physik 369: 253-287. https://doi.org/10.1002/andp.19213690304

[15] Darden T, York D, Pedersen L (1993) Particle mesh Ewald: An N·log(N) method for Ewald sums in large systems. The Journal of Chemical Physics 98: 10089-10092. https://doi.org/10.1063/1.464397

[16] Fick A (1855) V. On liquid diffusion. The London, Edinburgh, and Dublin Philosophical Magazine and Journal of Science 10(63): 30-39. 10.1080/14786445508641925

[17] Marrink SJ, Berendsen HJC (1994) Simulation of water transport through a lipid membrane. The Journal of Physical Chemistry 98: 4155-4168. https://doi.org/10.1021/j100066a040

[18] Marrink SJ, Berendsen HJC (1996) Permeation process of small molecules across lipid membranes studied by molecular dynamics simulations. In: The Journal of Physical Chemistry. Vol 100. American Chemical Society, pp 16729-16738

[19] Wang Y, Gallagher E, Jorgensen C, et al. (2019) An experimentally validated approach to calculate the blood-brain barrier permeability of small molecules. Scientific Reports 9. https://doi.org/10.1038/s41598-019-42272-0

[20] Jorgensen C, Ulmschneider MB, Searson PC (2022) Atomistic Model of Solute Transport across the Blood–Brain Barrier. ACS Omega 7(1): 1100-1112. https://doi.org/10.1021/acsomega.1c05679

[21] Jorgensen C, Troendle EP, Ulmschneider JP, Searson PC, Ulmschneider MB (2023) A least-squares-fitting procedure for an efficient preclinical ranking of passive transport across the blood–brain barrier endothelium. Journal of Computer-Aided Molecular Design 37(11): 537-549. 10.1007/s10822-023-00525-1

[22] Michaud-Agrawal N, Denning EJ, Woolf TB, Beckstein O (2011) MDAnalysis: A toolkit for the analysis of molecular dynamics simulations. Journal of Computational Chemistry 32(10): 2319-2327. https://doi.org/10.1002/jcc.21787
